# Genome sequence and characterization of *Streptomyces* phage Pablito, representing a new species within the genus *Janusvirus*

**DOI:** 10.1038/s41598-022-22784-y

**Published:** 2022-10-22

**Authors:** Véronique Ongenae, Joana Azeredo, Andrew M. Kropinski, Daniel Rozen, Ariane Briegel, Dennis Claessen

**Affiliations:** 1 Molecular Biotechnology, Institute of Biology, Leiden University, P.O. Box 9505, 2300 RA Leiden, The Netherlands; 2 Centre for Microbial Cell Biology, Leiden University, Leiden, The Netherlands; 3 Centre of Biological Engineering, University of Minho, Braga, Portugal; 4 Departments of Pathobiology and Food Science, University of Guelph, Guelph, ON N1G 2W1 Canada

**Keywords:** Phage biology, Microbiology, Microbial genetics

## Abstract

Streptomycetes are ubiquitous soil bacteria. Here we report the complete and annotated genome sequence and characterization of *Streptomyces* phage Pablito, isolated from a soil sample in Haarlem, the Netherlands using *Streptomyces lividans* as host. This phage was able to infect a diverse range of *Streptomyces* strains, but none belonging to the sister genus *Kitasatospora*. Phage Pablito has double-stranded DNA with a genome length of 49,581 base pairs encoding 76 putative proteins, of which 26 could be predicted. The presence of a serine integrase protein indicated the lysogenic nature of phage Pablito. The phage remained stable over a wide range of temperatures (25–45 °C) and at pH ≥ 7.0, but lost infectivity at temperatures above 55 °C or when the pH dropped below 6.0. This newly isolated phage is closely related to *Streptomyces* phage Janus and Hank144 and considered a new species classified in the genus *Janusvirus*, within the subfamily *Arquattrovirinae*.

## Introduction

*Streptomyces spp*. are Gram-positive, spore-forming bacteria that thrive in almost all soil environments. They are well-known for their ability to produce a wide array of clinically-relevant antibiotics, such as streptomycin, neomycin, chloramphenicol and many more^1–3^. Most research has been focused on the industrial use of these bacteria and their secretion of secondary metabolites^4,5^. However, it remains largely unknown how bacteriophages can recognize, attach and infect these multicellular bacteria. *Streptomyces* have an unusual lifecycle, which starts when spores encounter a favorable environment. This triggers germination and outgrowth into a so-called vegetative mycelium. After a period of vegetative growth, aerial hyphae are formed that can develop into chains of spores. These spores can be dispersed to more favorable environments to establish new colonies. Previous research has shown that *Streptomyces* phages can only infect young vegetative mycelia, but are unable to infect spores^6,7^. However, most bacteriophage research has been focused on unicellular bacteria, while the interactions and underlying mechanisms of bacteriophages infecting multicellular bacteria, like *Streptomyces,* remains largely unexplored. Besides the phage growth limitation system^8,9^, chemical defense^10^ and recently discovered cell wall-deficiency^11^, many more defense mechanisms against phage attack might be present in *Streptomyces*. In addition, relatively few *Streptomyces* phages have been properly sequenced and characterized in comparison to other genera. Therefore, it is important to isolate new phages infecting multicellular bacterial species, allowing us to expand our knowledge about the immense viral diversity.

In this paper, we describe a newly discovered Actinobacteriophage, called *Streptomyces* phage Pablito, which was isolated from a soil sample in the Netherlands. This phage belongs to the genus *Janusvirus*, within the subfamily *Arquattrovirinae*. Phage Pablito is a temperate phage with an icosahedral capsid head of 56.1 ± 1.3 nm and a long non-contractile tail of 163.5 ± 0.4 nm. Finally, we provide a whole genome analysis of this newly identified phage. Together, these results shed light on phage taxonomy and can expand the knowledge on phage-*Streptomyces* interactions in the future.

## Materials and methods

### Bacteriophage isolation

Phage Pablito was isolated from a soil sample obtained from the National Park Zuid-Kennemerland in the Netherlands (N52°23′31″, E4°34′49″). To this end, 0.5 g soil was mixed with spores from the host *S. lividans* strain 1326 in 10 ml Difco Nutrient Broth (DNB) (BD Biosciences) supplemented with 0.5% glucose and 4 mM Ca(NO_3_)_2_ and incubated overnight at 30 °C, while shaking at 130 RPM. After 24 h, the culture was centrifuged for 10 min at 4000 g and the supernatant containing phages was filtered through a 0.22 µm filter (Millipore). The supernatant was subsequently spread onto a DNB agar plate, followed by applying an overlay (in DNB soft agar) containing 10^3^ spores ml^−1^ from *S. lividans*. The plate was incubated at 30 °C for 24–48 h before plaque formation was assessed. Single plaques were purified by streaking them twice to a fresh double agar overlay plate containing the bacterial host. High titre single viral stocks were obtained by picking purified plaques and incubating them with *S. lividans* in DNB medium for 10 min before plating on double-agar overlay DNB plates. Viral lysates were collected by flooding the plate with 24 ml DNB and gently rocking the plate for 2 h. The supernatant was filtered through a 0.22 µm filter. Collected phages were stored at 4 °C.

### Host range analysis

The host range of phage Pablito was determined using serial dilutions on double agar overlay plates. In short, 3 µl of the phage dilution was spotted in duplicate on a bacterial lawn of different *Streptomyces* and *Kitasatospora* strains from the Microbial biotechnology (MBT) collection at Leiden University^12^, which represents an in-house collection of isolated actinomycetes. The plates were incubated at 30 °C for at least 24 h before lysis was observed. The presence of a lysis zone was scored as a positive result. The efficiency of plating (EOP) for a given strain was calculated relative to the host strain *S. lividans* when plaques were observed.

### Stability of phage Pablito

A one step-growth curve analysis was performed in triplicate to calculate the latent period and phage burst size^13^. In short, 10^5^ spores ml^−1^ of *S. lividans* were allowed to germinate in 10 ml DNB medium at 30 °C for 5 h. At *T* = 0, the culture was infected with phage Pablito at Multiplicity of Infection (MOI) = 0.01 and 100 µl was sampled at 10 min intervals up to 180 min. The samples were filtered, diluted and immediately plated on double agar plates. Burst size was calculated using the following formula:

$${\text{Burst}}\;{\text{size}} = \frac{{{\text{average}}\;{\text{of}}\;{\text{free}}\;{\text{phages}}\;{\text{after}}\;{\text{burst }}\left( {{\text{T}} = 90\;{\text{to}}\;{\text{T}} = 180} \right) - {\text{average}}\;{\text{of}}\;{\text{free}}\;{\text{phages}}\;{\text{before}}\;{\text{burst}}\;\left( {{\text{T}} = 10\;{\text{to}}\;{\text{T}} = 80} \right)}}{{{\text{number}}\;{\text{of}}\;{\text{phages}}\;{\text{at}}\;T = 0}}$$

The thermal stability of phage Pablito was determined by incubating 1 ml of lysate (10^6^ PFU ml^−1^) at 25, 30, 37, 45, 55 and 65 °C. The samples were filtered after 1 h of incubation and immediately plated on double agar plate to determine the phage titre. For pH stability, 10 µl of a phage Pablito suspension (10^6^ phages ml^−1^) was added to 990 µl DNB medium adjusted to different pH values (2–9) using 6 M HCl or 1 M NaOH. The tubes were then incubated at 30 °C for 1 h before the samples were filtered through a 0.22 µm filter and plated using the double agar overlay method. All measurements were performed in triplicate.

### DNA extraction and phylogenetic analysis

DNA of phage Pablito was isolated using the Phage DNA Isolation Kit from Norgen (Thorold, Canada) according to the manufacturers protocol. After isolation, multiple samples were pooled and DNA was concentrated using the Concentrator Plus (Eppendorf) for 20 min. Whole-genome sequencing and de novo assembly of phage Pablito was performed by BaseClear using Illumina NovaSeq PE150 sequencing with a mean coverage of 118.21 (Leiden, the Netherlands). The complete genome sequence of *Streptomyces* phage Pablito was deposited at GenBank under accession number OK412919. A linear map of the genome was constructed by Geneious Prime 2020.1.2 and PhageTerm was used to determine genome ends^14^.

Phylogenetic analysis was performed using the putative protein sequence of the major capsid head protein. The protein sequences of *Streptomyces* phage Janus, Hank144, Pablito and Joe were acquired from NCBI and aligned using MUSCLE^15^. A Maximum Likelihood phylogenetic tree was constructed through Molecular Evolutionary Genetics Analysis Version X (MEGAX) using 100 bootstrap replicates to estimate the genus of Phage Pablito.

### Electron microscopy

Two liquid cultures of *S. lividans* infected with phage Pablito were filtered, combined and precipitated overnight with 5 × PEG solution (20% PEG8000 in 2.5 M NaCl) at 4 °C and centrifuged for 70 min at 3500 g after which the supernatant was discarded. Of the remaining pellet, 3 µl was placed on a glow discharged 200 mesh carbon coated copper grid (EMS) and allowed to set for 30 s before excess sample solution was removed by filter paper. The phages were stained with 2% uranyl acetate for 45 s and air-dried for an additional 30 min. The grids were observed using a single tilt specimen holder inside a 120 kV Talos L120C TEM with a Lab6 electron source and Ceta detector at the Netherlands Center for Nanoscopy (NeCEN, Leiden).

## Results and discussion

### Morphology and host range determination of Pablito

**Figure 1:**
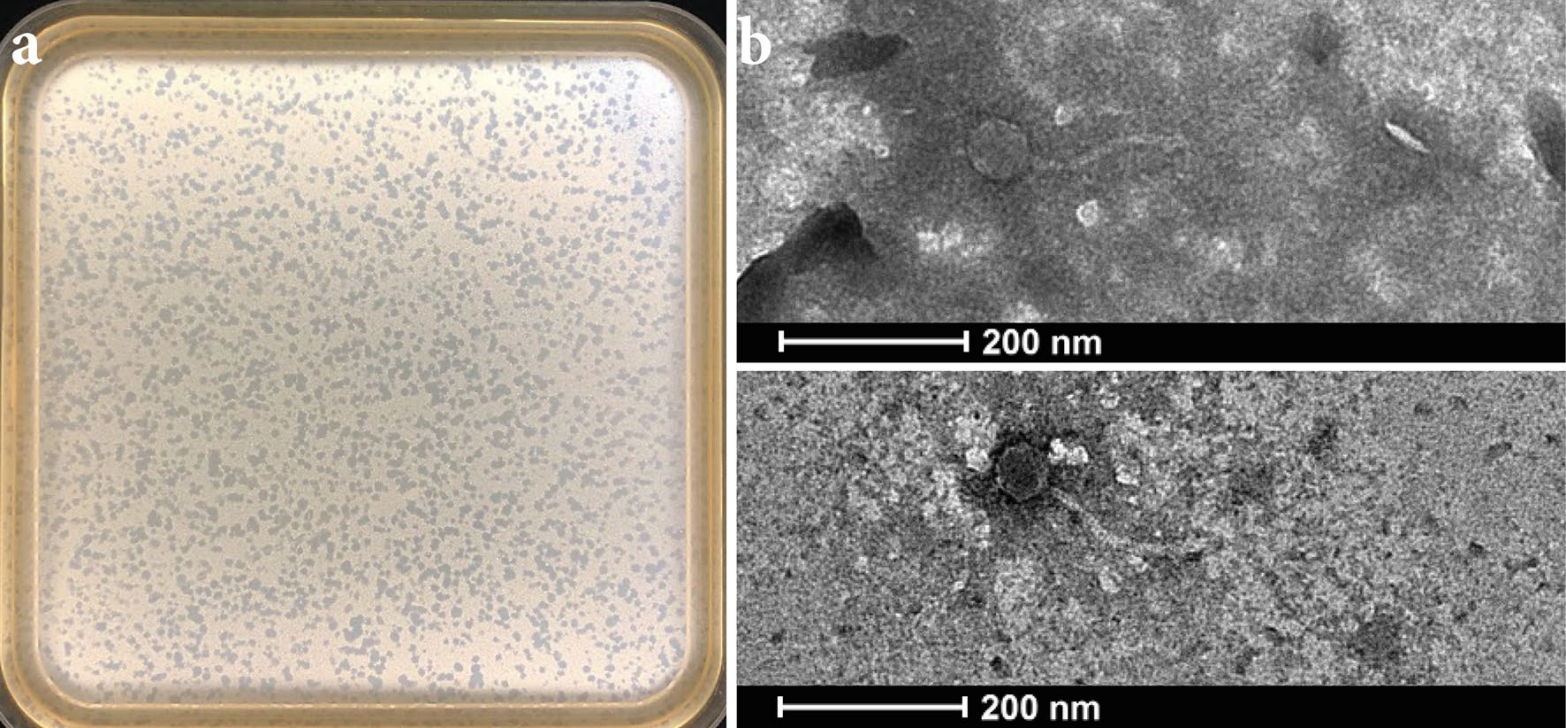
Morphology of phage Pablito. (**a**) Plaque morphology of phage Pablito after 24 h of infection in *S. lividans* using the DNB double agar overlay plate. (**b**) Representative TEM images of phage Pablito. Phage particles were stained with 2% uranyl acetate.

Bacteriophage Pablito produced clear small plaques on the *S. lividans* host strain using the double agar overlay method (Fig. 1a). Transmission electron microscopy (TEM) was used to reveal the morphology of phage Pablito. This demonstrated that phage Pablito has an icosahedral capsid head of 56.1 ± 1.3 nm (*n* = 3) and a long non-contractile tail of 163.5 ± 0.4 (*n* = 3) (Fig. 1b). The long, flexible tail together with the icosahedral capsid head were the first indication that this phage is a siphovirus belonging to the class *Caudoviricites*, which was further confirmed by whole-genome sequencing (see below).

A host range assay on 10 actinomycetes revealed that all eight *Streptomyces* strains tested could be infected by phage Pablito (Table 1). Depending on the host strain, some lysis zones showed a turbid morphology, which is common for several *Streptomyces* phages^16^, and was the first indication that phage Pablito might be a temperate phage. The EOP for a strain relative to the host strain *S. lividans* from which the phage was isolated, is given when plaques were countable. On the contrary, the two species that remained uninfected (i.e. MBT66 and *K. viridifaciens*) belong to the sister genus *Kitasatospora,* members of actinomycetes which are morphologically similar to *Streptomyces* but generally classified by their resistance against *Streptomyces* phages^17,18^.

**Table 1:** Susceptibility of actinomycete strains to infection by phage Pablito as determined by plaque assays.

Bacterial strain	Origin	Infectivity	Lysis zone morphology	EOP
*Streptomyces lividans*	MBT collection	+	Clear	1
*Streptomyces coelicolor*	MBT collection	+	Turbid	
*Streptomyces griseus*	MBT collection	+	Turbid	
*Streptomyces venezuelae*	MBT collection	+	Turbid	
*Streptomyces albus*	MBT collection	+	Clear	0.3
*Kitasatospora virifidaciens*	DSM40239	−	None	
MBT13	MBT collection	+	Turbid	
MBT61	MBT collection	+	Turbid	
MBT66	MBT collection	−	None	
MBT86	MBT collection	+	Clear	1.5

### Genomic analysis

**Figure 2:**
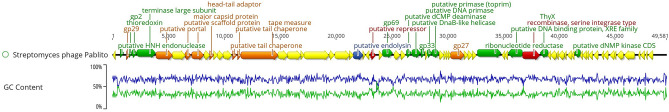
Schematic representation of the dsDNA genome of *Streptomyces* phage Pablito. Arrowheads are indicated in the direction of the CDS. Hypothetical proteins are displayed in yellow and the color code for predicted proteins is as follows: green for nucleotide metabolism and DNA replication, orange for morphogenesis, red for lysogeny and dark blue for bacterial lysis. The green line represents AT content, while the blue line represents GC content. The genome map was generated through Geneious Prime 2020 1.2.

Whole genome sequencing and de novo assembly of phage Pablito revealed a double-stranded DNA genome of 49,581 base pairs. While its host *S. lividans* has relatively high G + C content of 72%, *Streptomyces* phage Pablito has a G + C content of 66.4% and a 3′-cohesive termini of CGCCGTGTCTT (Fig. 2). The genome contains 76 potential coding sequences (CDSs), of which 50 encode for hypothetical proteins, while the function of 26 CDSs could be predicted. The latter were categorized as morphogenesis proteins, nucleotide metabolism and DNA replication proteins, lysogenic module and a bacterial lysis protein as indicated in Fig. 2.

**Figure 3:**
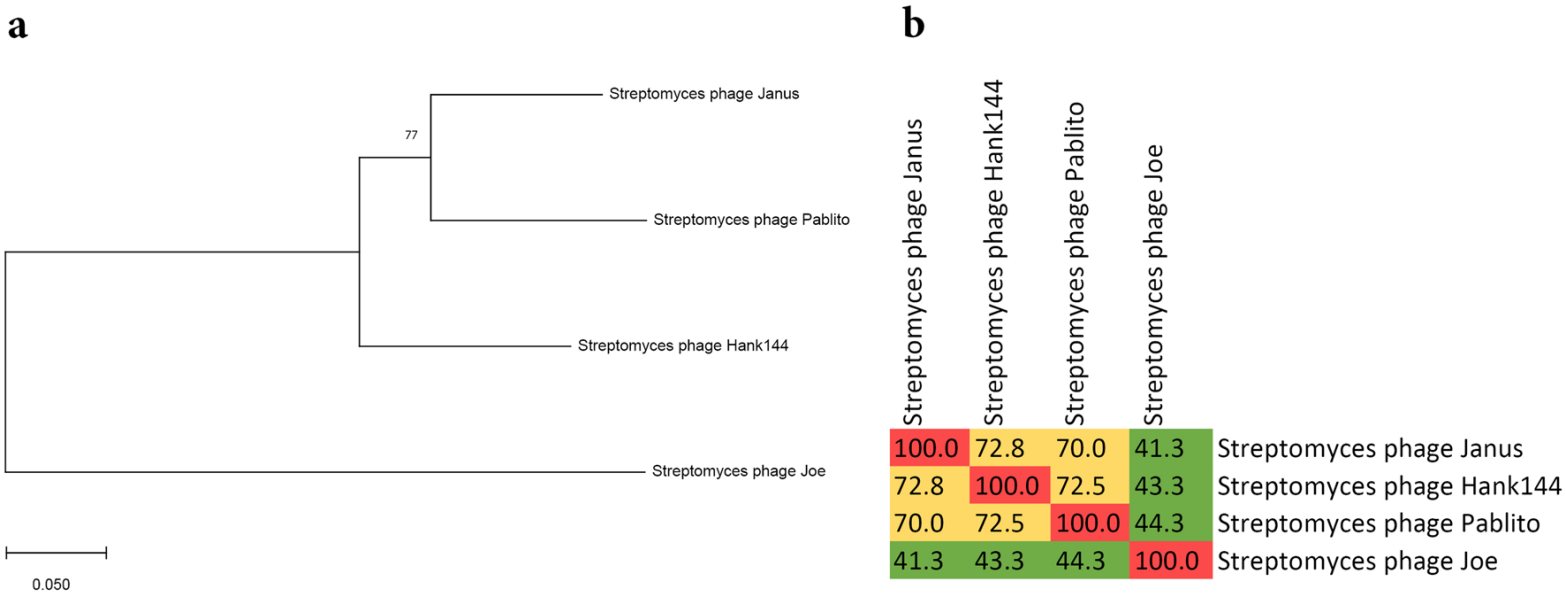
*Streptomyces* phage Pablito belongs to the subfamily *Arquattrovirinae*, genus *Janusvirus*. (**a**) Evolutionary history of phage Pablito based on the major capsid head protein. Total of 100 bootstraps replicates were used to calculate the Maximum-Likelihood tree in MEGA.X. (**b**) Heatmap calculated by VIRIDIC^20^ to show pairwise intergenomic distance/relatedness amongst phage genomes. Genomes of *Streptomyces* phage Janus (NC_054660.1) and *Streptomyces* phage Hank144 (NC_054661.1) were used to show relatedness, while the genome of *Streptomyces* phage Joe (NC_054674.1) was used as an outlier.

Phylogenetic analysis (Fig. 3a) based on the putative major capsid head protein revealed that *Streptomyces* phage Pablito is similar to *Streptomyces* phage Janus and *Streptomyces* phage Hank144, while *Streptomyces* phage Joe is more distantly related. Based on the criteria from the Bacterial Viruses Subcommittee, 70% nucleotide identity is established as the cut-off for genera, while 95% identity assigns phages to the same species^19^. As calculated by the intergenomic distance calculator VIRIDIC^20^, *Streptomyces* phage Pablito shows 70.0 and 72.5% relatedness to *Streptomyces* phages Janus and Hank144, respectively (Fig. 3b). Since the relatedness of *Streptomyces* phage Pablito is lower than 95%, this phage is considered a new species according to the International Committee on Taxonomy of Viruses (ICTV)^20^. Because *Streptomyces* phage Janus and *Streptomyces* phage Hank144 are both temperate phages and whole genome sequencing revealed the presence of a serine integrase protein in *Streptomyces* phage Pablito, we conclude that phage Pablito is a temperate phage as well^21^. Together, these data indicate that *Streptomyces* phage Pablito is a newly discovered species that can be included in the genus *Janusvirus*. A taxonomic proposal has been submitted to ICTV to officially recognize phage Pablito as part of the genus *Janusvirus* as a new species called “*Janusvirus Pablito*”. The Actinobacteriophage Database (https://phagesdb.org/) places phage Pablito in Cluster BD, Subcluster BD2. In addition, phage Pablito is peripherally related to two prophages found in *Streptomyces finlayi* strain NBSH44 and *Streptomyces venezuelae* strain ATCC 14583.

### One-step growth curve

**Figure 4:**
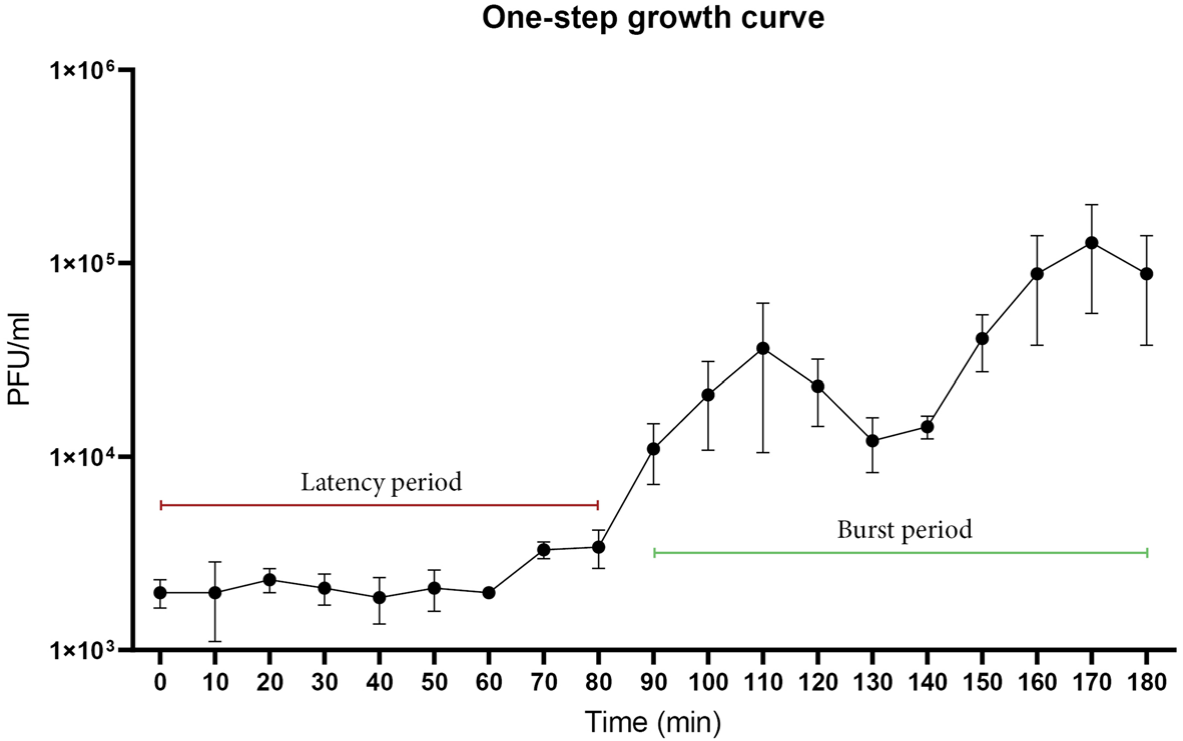
One-step growth curve. At MOI = 0.01, phage Pablito had a latent period of 80 min and a second burst at 150 min with a total burst size of 22 virions per cell.

*Streptomyces* phage adsorption was found to be maximal for germlings^16^. We therefore incubated *S. lividans* spores first for 5 h in DNB medium before infecting the culture with bacteriophages. The one-step growth curve of phage Pablito showed a relatively long latent period of 80 min, with a burst size of 22 virions per cell (Fig. 4). However, another rise of viral titer was observed around 150 min, which can happen when multiple phages bind to one germinated spore, as a sign of delayed adsorption or as an indication of a lysogenic infection cycle^22^. The highest titre obtained was around 10^6^ PFU ml^−1^ after 24 h. A different range of MOI, longer incubation periods and even flooding of a confluent plaque plate did not result in an increase of the total number of phages ml^−1^.

### Phage stability

**Figure 5:**
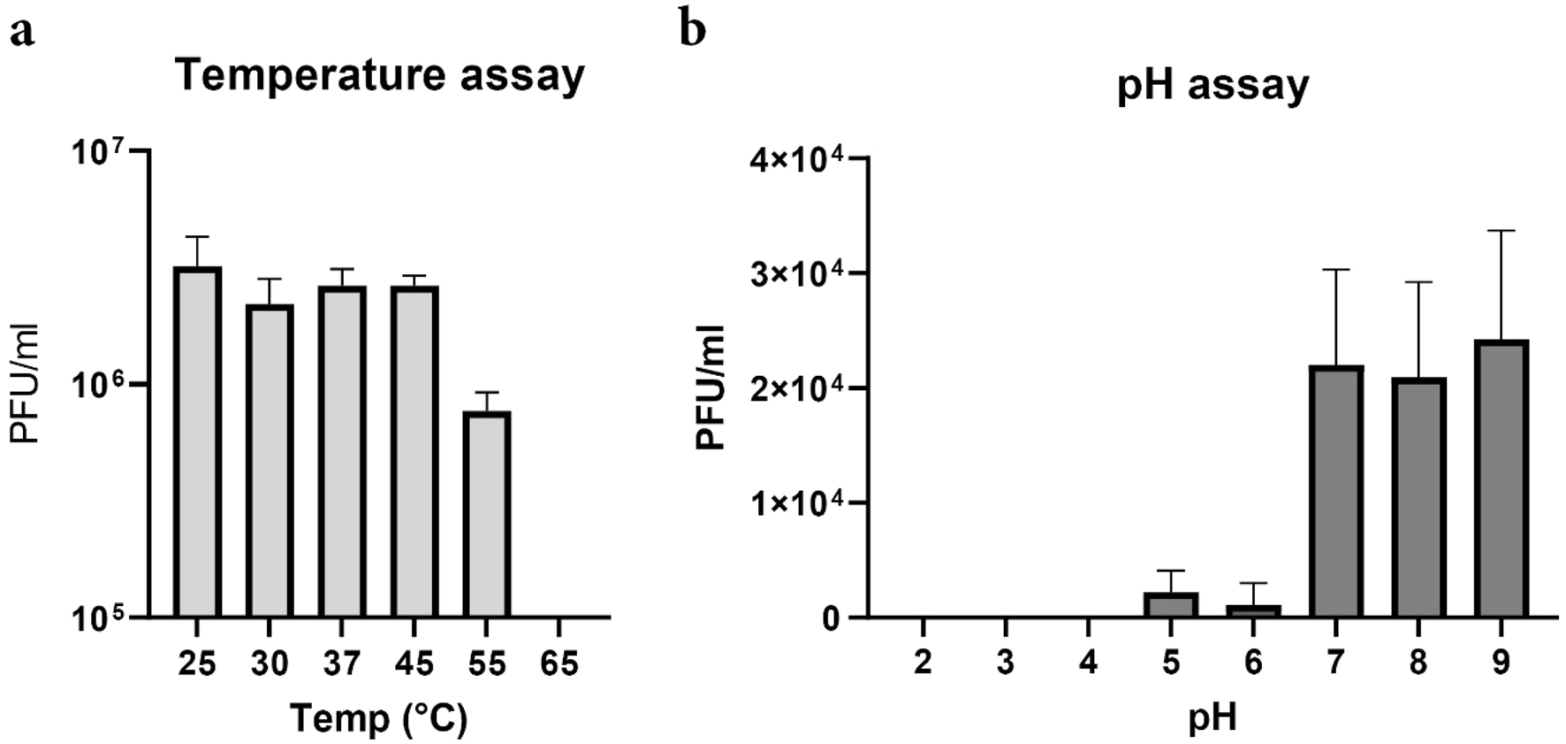
Stability of phage Pablito. (**a**) Phage Pablito remains relatively stable at temperatures ranging from 25 to 45 °C, but decreased stability is observed at higher temperatures. (**b**) Phage stability was optimal at pH of 7.0 and higher. A significant drop in PFU’s was observed at pH ≤ 6.0.

We also assessed the effect of temperature and pH on phage viability. Phage Pablito was stable up to 45 °C without a reduction in viability (Fig. 5a). However, the infectivity significantly decreased when the phage was incubated for an hour at 55 °C or higher. Phage Pablito was relatively stable at pH 7.0–9.0, while infectivity steeply declined when the pH was lower than 6.0 (Fig. 5b). No plaques were found when the pH was below 4.0, while only a small amount of activity was retained at pH 5.0 and pH 6.0.

## Conclusion

This study presents *Streptomyces* phage Pablito as a temperate siphophage being able to infect diverse *Streptomyces* strains. Genomic analysis and TEM images showed that this phage could be classified as a new species in the genus *Janusvirus* within the subfamily *Arquattrovirinae*. Members of this subfamily/genus have long non-contractile tails and icosahedral capsid heads. Phage Pablito showed a burst size of 22 virions per cell and was stable at temperatures ranging from 25 to 45 °C and a pH between 7.0 and 9.0. This phage can be used in future studies to shed new light on bacteriophage-*Streptomyces* interactions and disclose how this phage affects soil community.

## Data Availability

The genome of *Streptomyces* phage Pablito is available on NCBI (GenBank Accession No. OK412919).
